# Aqueous extract of *Lithospermi* radix attenuates oxaliplatin-induced neurotoxicity in both in vitro and in vivo models

**DOI:** 10.1186/s12906-016-1396-2

**Published:** 2016-10-26

**Authors:** Eun-Sang Cho, Jin-Mu Yi, Jong-Shik Park, You Jin Lee, Chae Jun Lim, Ok-Sun Bang, No Soo Kim

**Affiliations:** 1 KM-Convergence Research Division, Korea Institute of Oriental Medicine, 1672 Yuseong-daero, Yuseong-gu, Daejeon 34054 Republic of Korea; 2 Department of Korean Medicine, Life Science and Technology, Korea University of Science and Technology, Daejeon, Republic of Korea; 3 Current address: Department of Pathology, Chronic Inhalation Toxicity Research Center, Chemicals Toxicity Research Bureau, Occupational Safety and Health Research Institute, Korea Occupational Safety and Health Agency, Daejeon, Republic of Korea; 4 Current address: New Drug Development Center, K-BIO Osong Medical Innovation Foundation, Cheongju, Republic of Korea

**Keywords:** *Lithospermi* radix, Oxaliplatin, Peripheral neuropathy, Mechanical hypersensitivity, Inflammation

## Abstract

**Background:**

Oxaliplatin can induce peripheral neuropathy (OXIPN) as an adverse side effect in cancer patients. Until now, no effective preventive or therapeutic drug has been developed; therefore, the dose-limiting factor of OXIPN is still an obstacle in the use of oxaliplatin to treat cancer patients. In the present study, we report for the first time that the aqueous extract of *Lithospermi* radix (WLR) can attenuate the OXIPN in both in vitro and in vivo neuropathic models.

**Methods:**

The protective effect of WLR on OXIPN was evaluated in vitro by quantifying nerve growth factor (NGF)-stimulated neurite outgrowth in PC12 cells treated with a combination of oxaliplatin and WLR. The neuroprotective potential of WLR was further confirmed by measuring the changes in nociceptive sensitivities to external mechanical stimuli in neuropathic animals induced by oxaliplatin. Histological and immunohistochemical studies were further done to examine the effect of WLR in mouse spinal cords and footpads.

**Results:**

Oxaliplatin-induced neurotoxicity in NGF-stimulated PC12 cells. It could reduce the lengths and branching numbers of neuritis in NGF-stimulated PC12 cells. Co-treatment of WLR rescued the differentiated PC12 cells from the neurotoxicity of oxaliplatin. In a chronic OXIPN animal model, administration of oxaliplatin i.p. induced enhanced nociceptive sensitivity to mechanical stimuli (25.0 to 72.5 % of response rate) along with spinal activation of microglias and astrocytes and loss of intraepidermal nerve fibers in footpads, which is remarkably suppressed by oral administration of WLR (67.5 to 35 % of response rate at the end of experiment). Cytotoxicity of oxaliplatin determined in human cancer cells was not affected irrespective of the presence of WLR.

**Conclusions:**

In conclusion, we demonstrated that WLR can attenuate OXIPN in both in vitro and in vivo experimental models, which may be in part attributed to its anti-inflammatory activity in the spinal cord and its neuroprotective potential in the peripheral nerve system without affecting the anti-tumor potential of oxaliplatin. Therefore, WLR could be considered as a good starting material to develop a novel therapeutic agent targeting OXIPN. However, further studies should be done to elucidate the underlying mechanism such as molecular targets and active constituent(s) in WLR with neuroprotective potential.

## Background

Cancer has long been the leading cause of death in the Republic of Korea. The National Cancer Information Center reported that the cancer incidence in the Republic of Korea was 285.7 per 100,000 in 2013, which is higher than the average (270.3) of the OECD countries [1]. More than 76,000 people died from cancer and colorectal cancer (CRC) was ranked the 4^th^ accounting for 16.5 deaths per 100,000 people in 2014 [2]. CRC is occasionally considered as a disease of “developed countries” because the incidence of CRC is relatively higher among the countries with high-income economy than the other counterparts [3]. Since the western life style has prevailed among Koreans, the incidence of CRC has also increased continuously, and now, it has become one of the critical socio- economic burdens.

Oxaliplatin, a trade name for Eloxatin, is a third-generation platinum-based chemotherapeutic consisting of platinum coordinated with diaminocyclohexane and oxalate [4]. Oxaliplatin was the first platinum-based chemotherapeutic approved for advanced CRC and has been commonly used to treat CRC in combination with folinic acid and 5-fluorouracil known as FOLFOX [4, 5]. The cytotoxic activity of oxaliplatin is attributed to the formation of DNA adducts resulting from the intra- and inter-strand DNA crosslinks which inhibit DNA replication and transcription, followed by the generation of reactive oxygen species (ROS) and the ensuing DNA lesions and reduced glutathione depletion [6, 7]. Along with its pharmaceutical values in oncology, however, oxaliplatin is also known to induce adverse side effects which are related to its hematopoietic, gastrointestinal, and neuronal toxicities [4, 8, 9]. Oxaliplatin-induced peripheral neuropathy (OXIPN) is a member of a disease category called chemotherapy-induced peripheral neuropathy (CIPN), and is the most notorious, dose-limiting adverse side effect related with oxaliplatin [10]. Clinical symptoms of OXIPN are onset of pain, numbness, tingling, cramping, and hypersensitivity to cold but not to heat in the hands or feet [11–13]. It was reported that almost 50 % of cancer patients receiving a cumulative dose of oxaliplatin of 1,000 mg/m^2^ or higher, which is the standard treatment protocol, experience OXIPN-related symptoms [10], 40 % of patients continue to experience neuropathy for six months or longer, and 13 % of patients discontinue chemotherapy due to neuropathy [14]. Unfortunately, however, the molecular mechanisms underlying development of CIPN including OXIPN have not been fully elucidated and no preventive or therapeutic agents for CIPN have been approved by the US FDA [15]. Therefore, only symptomatic managements are provided to CIPN patients in clinics [16]. As diagnostic technologies of cancer are getting more advanced and the structure of the world population moves fast towards aged societies, it is evident that the number of cancer patients receiving chemotherapy will increase and accordingly, the number of patients suffering from CIPN as well. Therefore, the development of novel efficient preventive or therapeutic agents targeting CIPN still remains a major medical unmet need in oncology.

In our preliminary study, we tried to find effective materials to relieve oxaliplatin-mediated neurotoxicity using a library of medicinal herb extracts that have been traditionally used. We screened extracts with biological activities to relieve oxaliplatin-mediated neurotoxicity using in vitro cell-based assays which employed the nerve growth factor (NGF)-stimulated neurite growth from rat pheochromocytoma PC12 cells, and found that the aqueous extract of *Lithospermi* radix (WLR) could effectively rescue the cells from the neurotoxicity of oxaliplatin. In the present study we further evaluated the in vivo efficacies of WLR using a neuropathic animal model, which suggested the possible mechanism underlying its neuroprotective potential. To our knowledge this is the first study reporting that WLR can efficiently attenuate the oxaliplatin-induced neurotoxicity in both in vitro and in vivo models without affecting the cytotoxicity profiles of oxaliplatin.

## Methods

### Plant materials and WLR preparation

The dried *Lithospermi* radix was purchased from Kwangmyungdang Medicinal Herbs Co. (Ulsan, Republic of Korea) which had been collected from the area of Cheongsong-gun, Gyeongsangbuk-do, Republic of Korea in March 2015. The information of collection time and site was written on the batch label. It was identified by Dr. Go Ya Choi, at the K-herb Research Center, Korea Institute of Oriental Medicine, Republic of Korea. A voucher specimen (KIOM-CRC#187) was deposited at the KM Convergence Research Division, Korea Institute of Oriental Medicine, Republic of Korea. The powder of the plant material (100.0 g) was extracted twice in 2 L distilled water using a reflux extraction system at 100 °C for 2 h. The extract was filtered through Whatman filter paper No. 2 (Whatman International, Maidstone, UK), concentrated at 40 °C using a rotary evaporation system (N-1200A, EYELA, Tokyo Rikakikai, Tokyo, Japan), and freeze-dried using a freeze dryer (FD8518, IlshinBioBase, Dongduchun, Republic of Korea) to produce a soluble-aqueous extract (WLR, 35.2 % yield). The freeze-dried WLR was homogenized, stored in a tight-sealed bottle containing silica gel pouches, and kept in a refrigerator with light protection. For the in vitro studies WLR was dissolved in phosphate-buffered saline (PBS), sterilized using a 0.22 μm syringe filter (Satorius, Goettingen, Germany), aliquoted in a small volume, and then stored at −80 °C until used. For the animal studies, WLR was suspended in 0.5 % (w/v) carboxymethylcellulose (CMC, Sigma, St. Louis, MO, USA) dissolved in distilled water right before the experiments.

### In vitro cell culture

Rat pheochromocytoma PC12 cells (CCL-1721), human colorectal carcinoma HCT116 cells (CCL-247), and human lung carcinoma A549 cells (CCL-185) were obtained from the American Type Culture Collection (Rockville, MD, USA). Human neonatal foreskin fibroblast (HFFn, PC501A-HFF) cells were obtained from Systems Biosciences (Mountain View, CA, USA). PC12 cells were maintained on a 100 mm culture dish pre-coated with collagen type I (Corning, Kennebunk, ME, USA) containing DMEM supplemented with 10 % heat-inactivated horse serum, and 5 % non-heat-inactivated fetal bovine serum (FBS). A549, HCT116, and HFFn cells were maintained with RPMI1640, McCoy’s 5A, and DMEM, respectively, supplemented with 10 % heat-inactivated FBS. All culture media were supplemented with 100 U/mL penicillin and 100 μg/mL streptomycin to minimize microbial contamination. Basal media, serum, and antibiotics were purchased from Thermo Fisher Scientific (Waltham, MA, USA). Cells were maintained at 37 °C in a humidified incubator aerated with 5 % CO_2_.

### Cell viability

Cells were seeded at a density of 1,000 (HFFn) or 5,000 (HCT116 and A549) cells/well into a 96-well tissue culture plate and cultured overnight. The cells were exposed to drugs for 48 h and then, cell viabilities were determined with the EZ-Cytox cell viability assay kit (Daeil Lab Service, Seoul, Republic of Korea) measuring viable cells with normal mitochondrial function as described in the manufacturer’s instructions. The development of color was monitored and quantified at 450 nm using the Emax microplate reader (Molecular Devices, Sunnyvale, CA, USA). The relative viabilities were determined by comparing the color intensity of the drug treatment with those of the vehicle treatment. The half maximal inhibitory concentration (IC_50_) was calculated with the SoftMax Pro 4.0 software (Molecular Devices).

### Neurite outgrowth assay

PC12 cells (1 × 10^4^ cells/well) were plated on a 24-well tissue culture plate pre-coated with collagen type IV (Corning) containing a complete culture medium. After 24 h, the medium was replaced with serum free DMEM containing 100 ng/mL recombinant rat beta-NGF (R&D systems, Cat # 556-NG, Minneapolis, MN, USA), N2 supplement (Thermo Fisher Scientific, Cat # 17502048) and 0.5 % FBS to induce neurite growth in the presence or absence of oxaliplatin (200 nM, LC Laboratories, Woburn, MA, USA). WLR (25 and 100 μg/mL) or amifostine (0.5 mM, Santa Cruz Biotechnology, Dallas, TX, USA) were co-treated with oxaliplatin. Three days after the drug treatment, a morphometric analysis was done on digitalized images of PC12 cells taken with an inverted microscope (IX71, Olympus, Center Valley, PA, USA). Images of fields with more than 20 cells were captured and used for the image analysis. The differentiation of cells induced by NGF was examined, and cells that had at least one neurite with a length equal to the cell body diameter were counted. The number of neurite bearing cells was expressed as a percentage of the total cells. Total neurite length was determined by manually tracing the length of the neurite per cell using the MetaMorph image software (Molecular Devices).

### Experimental animals

Eight-week-old male C57BL/6 mice were obtained from OrientBio Inc (Seongnam, Republic of Korea) and maintained in a specific-pathogen-free laboratory animal care facility. Animals were housed in a temperature (22 ± 2 °C)- and humidity (45 ± 10 %)-controlled vivarium (12/12-h dark/light cycle with lights on at 8 a.m.) with free access to food and water. Animals were acclimated for 1 week before the experiments. All procedures for animal care and experiments were reviewed and approved by the Korea Institute of Oriental Medicine Institutional Animal Care and Use Committee.

### Induction of peripheral neuropathy in animals

Oxaliplatin was dissolved in a physiological saline solution (Samyang Anipharm, Seoul, Republic of Korea) and administered intraperitoneally (i.p.) at dose of 5 mg/kg. A week later, another dose of oxaliplatin (5 mg/kg, i.p.) was given to induce peripheral neuropathy (total 10 mg/kg). Control mice received the same volume of saline as oxaliplatin treatment.

### Drug administration

One week after the final oxaliplatin injection, animals were divided into 3 groups, and each animal group received WLR (250 mg/kg in 0.5 % (w/v) CMC, oral, 6 days per week, *n* = 8), vehicle (0.5 % CMC, oral, 6 days per week, *n* = 8), or amifostine (100 mg/kg in 0.9 % saline, intraperitoneal, once a week, *n* = 10) between 9–10 a.m. for 4 weeks. For comparison, saline control mice (i.e., no oxaliplatin treatment, *n* = 8) received 0.9 % saline (vehicle for oxaliplatin) and 0.5 % CMC (vehicle for WLR). In pharmacopoeia of Korean traditional medicine, the *Lithospermi* radix has been usually prescribed for nourishment of blood, enhancement of blood stream, detoxification, anti-inflammation, skin damage, etc. In those cases, decoction of 3–10 g of *Lithospermi* radix is recommended as a daily dosage. However, there was no record of *Lithospermi* radix for relieving or ameliorating the neuronal pain. Therefore, we used the recommended doses of *Lithospermi* radix for other cases to evaluate its protective effect against OXIPN. In our lab, the yield of *Lithospermi* radix extract in hot water (WLR) was almost 40 % and we could expect to get 1.2-4 g of WLR from 3–10 g of raw plant materials. On the assumption that the average body weight of healthy adults is 60 kg, the daily doses of WLR is 20–67 mg/kg. So, we chose 250 mg/kg for initial dose for our animal studies using mice, which is equivalent to 20 mg/kg in adult humans [17].

### Behavioral tests

The sensitivity of experimental animals to external mechanical stimuli were determined by an operator blinded to the status of the drug treatment in a quiet, temperature (22 ± 2 °C)-controlled behavior test room between 1–5 PM. Mice were individually placed in a black plastic chamber (5 × 5 × 8 cm) set on the perforated metal mesh-like open grid of square holes (5 × 5 mm). Animals were acclimated for at least 30 min before the behavior test. Mechanical sensitivity was determined using a calibrated von Frey filament with 0.4 g bending force (Aesthesio, BanMic Global, LLC, San Jose., CA, USA). The filament was applied to the plantar surface of the right hind paw for 1 s with an interval of 5 s between stimulations. The test was repeated 5 times with an interval of at least 5 min between each test cycle. The frequencies of paw withdrawal were calculated as a percentage of response (rapid and sudden lifting, shaking, or licking) occurring at least 3 times from a total of 5 trials.

### Immunohistochemistry

Four weeks after the last oxaliplatin injection, the mice were deeply anesthetized by intraperitoneal injection of sodium pentobarbital (50 mg/kg, Hanlim Pharmaceuticals Inc, Seoul, Republic of Korea) and transcardially perfused with 4 % (w/v) paraformaldehyde prepared in 0.1 M PBS, pH 7.4). After perfusion, the L4-L5 spinal cords and hind footpads were removed and post-fixed in the same fixative solution for immunohistochemical and morphometric analyses. The L4-L5 spinal cords were verified by identification of the lumbar enlargement and nerve roots. Immunohistochemical analyses were done with specific antibodies against glial fibrillary acidic protein (GFAP) and ionized calcium binding adaptor molecule 1 (IBA1) as markers of activated astrocytes and microglias, respectively, or with a specific antibody against tumor necrosis factor-alpha (TNF-α) as a marker of inflammation using the fixed spinal cords. Immunohistochemical analysis was also done with a specific antibody against protein gene product 9.5 (PGP9.5) as markers of intraepidermal nerve fibers (IENF) using the fixed hind footpads. The paraffin-embedded spinal cords and footpads were sectioned at a 4 μm thickness and then mounted on silane- coated slides (Muto Pure Chemicals, Tokyo, Japan). Sections were deparaffinized and immersed in unmasking solution (Vector laboratory, Burlingame, CA, USA) and microwaved for 10 min at 98 °C. After cooling, sections were washed in Tris-buffered saline with 0.5 % tween 20 (TBS-T) and then, incubated with primary antibody solutions (mouse monoclonal anti-GFAP, 1:200; rabbit polyclonal anti-PGP9.5, 1:200, Millipore, Temecula, CA, USA; mouse monoclonal anti-TNF-α, 1:200, Abcam, Carlsbad, CA, USA; rabbit polyclonal anti-IBA1, 1:200, WAKO, Wako, Osaka, Japan) at 4 °C overnight. After 3 washes with TBS-T, sections were incubated in a secondary antibody labeled with Alexa Fluor 488 and 548 (1:2,000, Abcam) for 1 h at room temperature. After washing 3 times with TBS-T, sections were covered with an aqueous mounting medium (Vector shield, Vector laboratory), examined, and photographed under a fluorescence microscope (BX41, Olympus, 200X for GFAP and IBAI, 400X for TNF-α and PGP9.5). The images were optimized using an image analyzing software (Cellsense, Olympus). The densities of the immunoreactive GFAP and IBA1 antibody in the superficial dorsal horn laminae were determined with Image J (v1.43, NIH, USA) and expressed as a percentage of the immunostained area (% area) compared to the total image area. Quantification of IENFs in the foot pad was conducted as follows; 5 randomly chosen slices per animal were analyzed using a fluorescence microscope. Nerve fibers that perpendicularly penetrated into the epidermis from dermal/epidermal junction were counted in 3 fields of view from each slice. The length of the epidermis within each field of view was measured using the Cellsense image analyzing software. The IENF density was determined as the total number of fibers per field length of epidermis that branched after crossing the basement membrane (IENF/mm) as previously described [18].

### Morphometric analysis on the dorsal root ganglia

Dorsal root ganglions (DRGs) were obtained from L4-L5 and fixed as described above. Post-fixed DRGs were embedded in paraffin, sliced at a 4 μm thickness, mounted on charged slides, and subjected to Azan-Mallory staining (Azan Trichrome kit, Gennova Scientific, Sevilla, Spain) as described in the manufacturer’s instructions. The numbers of DRG neurons with multiple and/or eccentric nucleoli were counted at a high magnification (400X). The nucleolus was classified eccentric when located in the outer half of the radius of the nucleus, and the cell was classified multinucleolated when it showed more than 1 nucleolus in the cell body. At least 5 sections for each animal were analyzed, and the values were expressed as a percentage of DRG neurons with multiple and/or eccentric nucleoli compared to the total DRG neurons.

### Statistical analysis

All statistical analyses were performed with SigmaPlot (v11.0, Systat Software, San Jose, CA, USA). The results are presented as the mean ± standard deviation (SD) of at least triplicate experiments. Differences were compared using one-way analysis of variance (ANOVA, parametric) or Kruskal-Wallis one-way ANOVA on ranks (nonparametric) according to Shapiro-Wilk normality test followed by the Tukey *post-hoc* test. The values with *p* < 0.05 were considered significant.

## Results

### Preventive effect of WLR on oxaliplatin-induced neurotoxicity in vitro

**Fig. 1:**
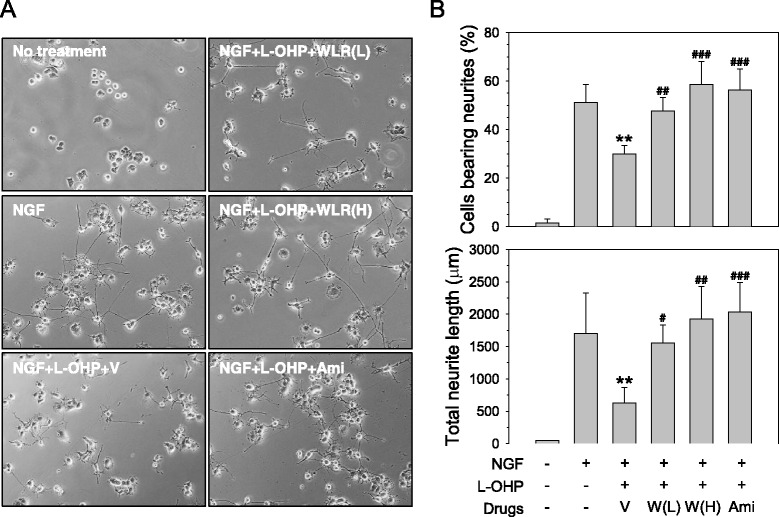
Protection from oxaliplatin-mediated neurotoxicity by WLR in differentiated PC12 cells. **a** PC12 cells were cultured on a collagen type IV-coated surface and neurite growth was initiated by the treatment of 100 ng/mL NGF. A combination of oxaliplatin (L-OHP, 200 nM) and WLR (L, 25 μg/mL; H, 100 μg/mL) were applied to the cell culture. Amifostine (Ami, 0.5 mM) and vehicle (V, PBS) were also included in parallel as positive and negative controls, respectively. Following 3 days of differentiation, the neurites sprouting from the cell bodies were observed under an inverted microscope (X100). **b** The percentages of cells bearing neurites and total neurite lengths were quantified using an image analyzing program. The data represent the combined results of *n* = 3 experiments. **p < 0.01 vs. only NGF-treated group (NGF); ^#^ *p* < 0.05, ^##^ *p* < 0.01, ^###^ *p* < 0.001 vs. NGF, L-OHP, and vehicle-treated group (NGF + L-OHP + V)

Oxaliplatin was previously reported to inhibit NGF-stimulated neurite outgrowth in PC12 rat pheochromocytoma cells [19]. The effect of WLR on oxaliplatin-induced neurotoxicity was evaluated using the neurite outgrowth assay involving neuronal differentiation of PC12 cells by NGF. PC12 cells without NFG (No treatment) had a round-shaped morphology and remained undifferentiated throughout the cell culture. Following NGF treatment, extensive neurites spread out from the cell bodies and formed neurite networks (Fig. 1a). The percentage of PC12 cells bearing neurites and total neurite length reached 51 % and 1703 μm, respectively (NGF, Fig. 1b). However, oxaliplatin significantly inhibited NGF-stimulated neurite outgrowth, and the percentage of PC12 cells bearing neurites and total neurite length were reduced to 30 % and 627 μm, respectively (NGF + L-OHP + V). Simultaneous treatment of WLR recovered cells from oxaliplatin-induced neurotoxicity in a dose-dependent manner, and the percentages of cells bearing neurites increased up to 48 % at 25 μg/mL WLR (NGF + L-OHP + WLR(L)) and 59 % at 100 μg/mL WLR (NGF + L-OHP + WLR(H)). The total neurite length also increased up to 1552 μm at 25 μg/mL WLR and 1924 μm at 100 μg/mL WLR, which were comparable with the NGF only-treated group (NGF). Amifostine (NGF + L-OHP + Ami), which was used as a positive control, recovered cells from oxaliplatin-induced toxicity with significant increases in the percentage of cells bearing neurites (56 %) and the total neurite length (2032 μm), which are consistent with a previous report by Ceresa et al. [20].

### Preventive effects of WLR on oxaliplatin-induced mechanical hypersensitivity

**Fig. 2:**
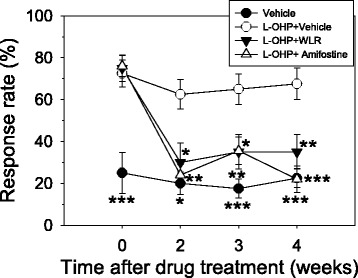
Progressive time course effect of daily administration of WLR on the nociceptive behavior of animals subjected to oxaliplatin-induced neurotoxicity. To induce OXIPN, animals received 5 mg/kg of oxaliplatin once per week for 2 weeks (total accumulated dose of 10 mg/kg). One week after the final oxaliplatin injection, WLR or amifostine was administered to the animals for 4 weeks as per dosing schedule. At regular time points, a mechanical stimulus was applied on the plantar surface of the right hind paw of the animals using a von Frey monofilament with a 0.4 g bending force, and the response rates were scored. The data represent the combined results of the oxaliplatin plus vehicle (0.5 % CMC, open circles, n = 8), plus WLR (250 mg/kg, closed triangles, n = 8), or plus amifostine (100 mg/kg, open triangles, n = 10) mice. Only the vehicle (0.9 % saline and 0.5 % CMC)-treated mice (closed circles, n = 8) were also included in parallel as a saline control group. *p < 0.05, **p < 0.01, ***p < 0.001 vs. saline-treated control group

The protective effect of WLR against oxaliplatin-induced CIPN were evaluated with peripheral neuropathy animal model which was induced by 2 times intraperitoneal injections of low dose oxaliplatin (accumulated dose of 10 mg/kg). WLR or vehicle were administered to animals as described earlier. Any clinical signs related with toxicity such as remarkable loss of body weight were monitored in all groups of animals during the course of experiment. The effect of WLR on mechanical hypersensitivity in mice induced by oxaliplatin injection is shown in Fig. 2. When von Frey filament with a 0.4 g bending force was applied to the plantar surface of the hind paw, the saline control mice that received 0.9 % saline and 0.5 % CMC showed 17–25 % response rates throughout the experiment. The mechanical sensitivity of mice that received oxaliplatin and 0.5 % CMC was enhanced, and the response rate was increased to 73 % 1 week after the final oxaliplatin injection (0 week) and maintained between 63–67 % until the experiment ended. Daily oral administration of WLR remarkably attenuated the oxaliplatin-induced mechanical hypersensitivity, and the response rate of mice was significantly decreased to 30 % two weeks after starting the WLR administration and then, maintained around 35 % until the experiment ended. Intraperitoneal administration of amifostine, a positive control drug that has been known to attenuate the platinum-based chemotherapy-induced mechanical hypersensitivity in both in vitro [21] and in vivo models [22], also remarkably attenuated the oxaliplatin-induced mechanical hypersensitivity, and the response rate was significantly decreased to 24 % two weeks after starting the drug administration and maintained between 22–35 % until the experiment ended. The significant differences between WLR- and amifostine-treated groups were not observed throughout the experiment.

### Immunohistochemical analyses of spinal activation of glial cells

**Fig. 3:**
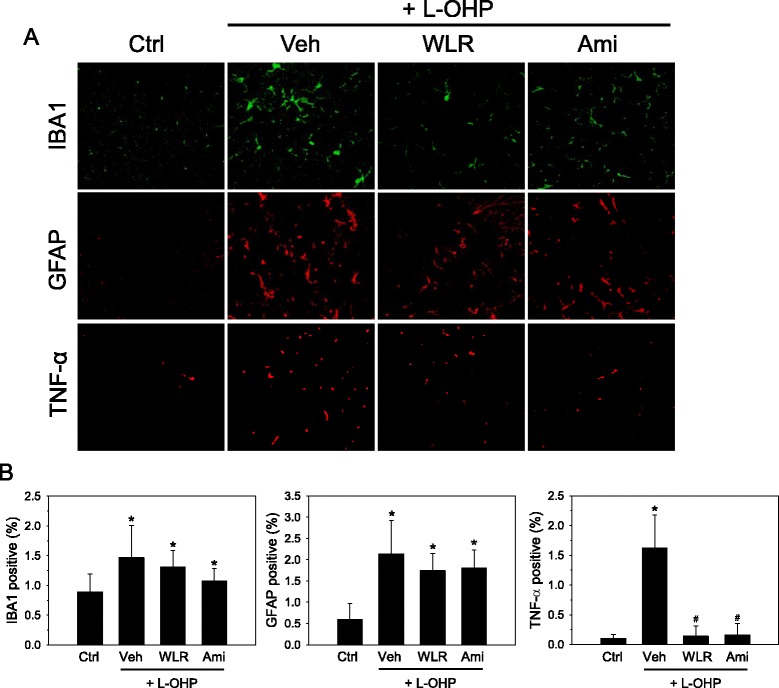
Anti-inflammatory effects of WLR in the spinal cords of the OXIPN animals. **a** Induction of neuropathy by oxaliplatin (L-OHP) and drug administration were done as described in Fig. 2. Four weeks after administration of WLR (250 mg/kg, 6 times a week) or amifostine (Ami, 100 mg/kg, once a week), the spinal cords of experimental animals were procured and subjected to immunohistochemical analyses. Microglias (green) and astrocytes (red) in the lumbar dorsal horn of the mouse spinal cords (L3-L4) were detected with IBA1 and GFAP specific antibodies, respectively (X200). Activated microglias and astrocytes showed marked hypertrophy in the oxaliplatin-treated animals. **b** Activated astrocytes and microglial cells were quantified by measuring the corresponding IBA1 and GFAP immunoreactivities, respectively, and were expressed as a percentage of immune-positive areas per total image areas. *p < 0.05 vs saline control mice (Ctrl); ^#^ *p* < 0.05 vs. oxaliplatin (L-OHP) and vehicle (Veh)-treated mice. The data represent the combined results of *n* = 5 (IBA1 and GFAP) or n = 4 (TNF-α) experiments

To investigate the relationship between OXIPN and glial cell activation in the central nervous system glial cell reorganization in the dorsal horns of the mouse spinal cords was assessed at the end of the experiment. Microglias and astrocytes were specifically visualized by immunohistochemical staining with IBA1 and GFAP antibodies (200X, Fig. 3a), respectively, and their cell densities were quantified by image analysis (Fig. 3b). Following oxaliplatin administration, the numbers of activated microglias and astrocytes were increased in the dorsal horn of the mouse spinal cords (Fig. 3a, L-OHP + Veh), which is consistent with a previous report by Di Cesare Mannelli et al. showing that repeated administration of oxaliplatin up to an accumulated dose of 36 mg/kg in rats induced the activation of both microglias and astrocytes in the dorsal horns of the rat spinal cords [23]. Quantification of the digital images shows that the densities of IBA1 positive microglial cells (1.5 %) and GFAP positive astrocytes (2.1 %) in the mouse spinal cords were significantly increased after oxaliplatin administration when compared to those of saline control mice (Ctrl, 0.9 % for microglias and 0.6 % for astrocytes). The spinal activation of microglias and astrocytes by oxaliplatin was slightly suppressed by WLR (L-OHP + WLR) to 1.3 and 1.7 %, respectively. Amifostine treatment used as a positive control drug in the present study (L-OHP + Ami) also slightly suppressed the spinal activation of both microglias and astrocytes to 1.1 % and 1.8 %, respectively. Neuroinflammation following administration of oxaliplatin was confirmed by observing increased numbers of pro-inflammatory TNF-α positive cells in the spinal cords. Co-treatment of WLR or amifostine reduced successfully enhanced TNF-α expression by oxaliplatin (Fig. 3a and b).

### Immunohistochemical examination of IENF distribution

**Fig. 4:**
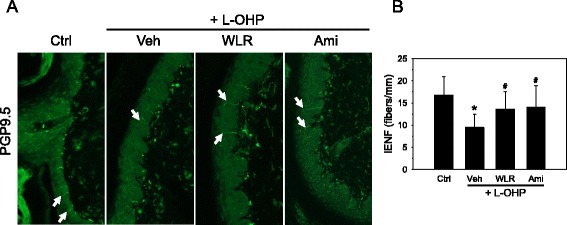
Neuroprotective effects of WLR in the peripheral nerves of OXIPN animals. **a** Induction of neuropathy by oxaliplatin (L-OHP) and drug administration were done as described in Fig. 2. Four weeks after administration of WLR (250 mg/kg, 6 times a week) or amifostine (Ami, 100 mg/kg, once a week), the footpads of the experimental animals were procured and subjected to immunohistochemical analyses. IENFs in the footpads were detected with the PGP9.5 specific antibody. IENFs that were immunostained with the pan-axonal marker PGP9.5 were vertically arrayed within the epidermis (arrows, X400). **b** Innervation of the IENFs through the mouse skins was quantified by scoring the IENFs within sections of the footpads and expressed as densities of IENFs (fibers/mm). *p < 0.05 vs saline control mice (Ctrl); ^#^ *p* < 0.05 vs. oxaliplatin (L-OHP) and vehicle (Veh)-treated mice. The data represent the combined results of *n* = 8 experiments

To understand the effects of oxaliplatin on the innervation of peripheral nerves in the distal foot skin, we evaluated the nociceptive nerve fibers in the epidermis defined as IENFs by immunostaining with the PGP9.5 specific antibody (Fig. 4a), and the density of the IENFs was quantified by digital analysis of the immunostained sections (Fig. 4b). In the saline control mice (Ctrl), the IENFs expressing PGP9.5 showed regular penetration through the epidermal-dermal junctions, and they were perpendicular to the epidermis. The density of the IENFs in the saline control mice, which was quantified by digital analysis of the immunostained sections, was 16.7 fibers/mm. After receiving oxaliplatin, loss of IENFs was observed (L-OHP + Veh), and the density of the IENFs was significantly decreased to 9.5 fibers/mm when compared to saline control mice. Such loss of IENFs by oxaliplatin could be recovered by daily oral administration of WLR (L-OHP + WLR), and the density of IENFs was significantly increased to 13.6 fibers/mm when compared to the oxaliplatin only-treated group (L-OHP + Veh). A significant reduction in oxaliplatin-induced IENF loss was also observed in mice treated with amifostine (L-OHP + Ami), and the density of IENFs was increased to 16.7 fibers/mm when compared with oxaliplatin alone. Mice receiving oxaliplatin with WLR or amifostine were not significantly different from the saline control mice in regards to IENF densities.

### Morphometric analysis of DRG neurons

**Fig. 5:**
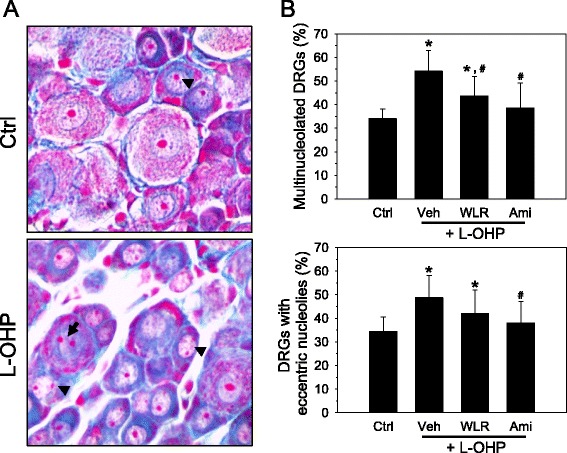
Neuroprotective effects of WLR in the DRGs of OXIPN animals. **a** Induction of neuropathy by oxaliplatin (L-OHP) and drug administration were done as described in Fig. 2. Four weeks after administration of WLR (250 mg/kg, 6 times a week) or amifostine (Ami, 100 mg/kg, once a week), DRG neurons from L4-L5 were procured and subjected to Azan-Mallory staining. The nuclei were observed at a high magnification (X400), and DRG neurons with nucleus containing atypical nucleoli were scored. When compared with the saline control mice, the DRGs of oxaliplatin (L-OHP)-treated mice showed an increased number of DRG neurons with eccentric nucleoli (arrowhead) and/or multinucleolated nuclei (arrows). **b** The neurons with multiple or eccentric nucleoli were counted and expressed as a percentage of the total neuron. *p < 0.05 vs saline control mice (Ctrl); ^#^ *p* < 0.05 vs. oxaliplatin (L-OHP) and vehicle (Veh)-treated mice. The data represent the combined results of *n* = 5 experiments

It was previously reported that the numbers of DRG neurons with atypical morphological nuclei, such as nucleolar eccentricity and/or multinucleolation, are increased during oxaliplatin-induced chronic neuropathy [23]. In the present study, the DRG neurons were subjected to Azan-Mallory staining, and the morphological changes in the DRG nuclei were observed under a light microscope to discern the relationship between OXIPN and neurotoxic damages in DRG neurons (Fig. 5a); thus, the percentage of DRG neurons with atypical nuclei was determined by image analysis (Fig. 5b). The DRG neurons from the oxaliplatin-treated mice (L-OHP + Veh) showed significantly increased numbers of nuclei with eccentric nucleoli (34.4 to 48.8 %) and multinucleolated neurons (34.0 to 54.2 %) when compared with the DRG neurons of saline control mice (Control). The percentage of DRG neurons of saline control mice with atypical nuclei in the present study was a little bit higher than those of normal Sprague-Dawley rats reported by Di Cesare Mannelli et al. with a 10–20 % nucleolar eccentricity and 20–30 % multinucleolated nuclei [23]. This may be due to variations among the different species of rodents. Daily oral administration of WLR (L-OHP + WLR) exerted a protective effect in the DRGs as evidenced by a significant reduction in the occurrence of multinucleolated DRG neurons (43.7 %) as well as a slight reduction in eccentric nucleoli (42.0 %) with borderline significance (*p* = 0.085). Amifostine treatment used as a positive control drug (L-OHP + Ami) also reduced the numbers of both multinucleolated DRG neurons (38.6 %) and eccentric nucleoli (38.1 %) with statistical significances.

### Determination of WLR toxicity

**Fig. 6:**
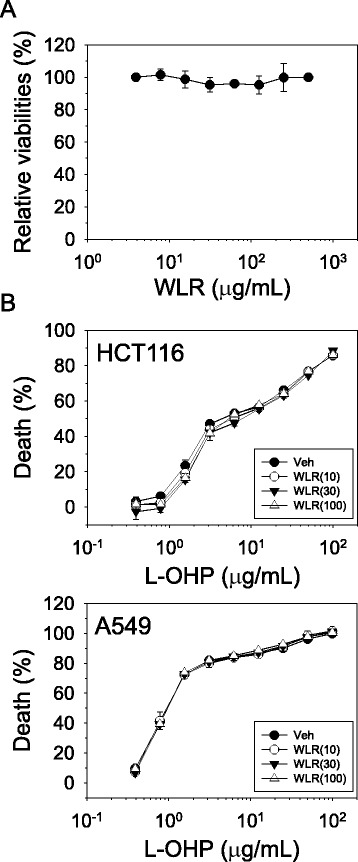
Effects of WLR on normal cell proliferation and on oxaliplatin-mediated cytotoxicity in cancer cells. **a** Human normal HFFn cells were subjected to increasing doses of WLR for 48 h, and then, cell viability was determined as described earlier. Relative viability was determined by comparison with the vehicle (PBS) treatment. **b** The effect of WLR on oxaliplatin-mediated cytotoxicity was evaluated in HCT116 human colorectal cancer cells and A549 human lung cancer cells. Both cells were subjected to a combination of increasing doses of oxaliplatin (0–100 μg/mL) and WLR (PBS, 10, 30, 100 μg/mL) for 48 h, and then, cell viability was determined. The data represent the combined results of *n* = 3 experiments

The toxicity of WLR itself was evaluated using human normal neonatal foreskin fibroblast (HFFn). As shown in Fig. 6a, the cell viability was maintained higher than 95 % even in the presence of WLR up to 500 μg/mL.

### Effects of WLR on the anti-tumor activity of oxaliplatin

The effects of WLR on the anti-tumor activity of oxaliplatin were assessed in HCT116 (colorectal) and A549 (lung). Figure 6b presents the mean deaths of HCT116 (upper panel) and A549 (lower panel) cells after co-treatment of oxaliplatin (0–100 μg/mL) and WLR (0–100 μg/mL) for 48 h. The toxicity profiles of oxaliplatin in HCT116 and A549 cells were similar irrespective of the WLR concentrations. The IC_50_s of oxaliplatin in HCT116 and A549 in the presence of varying concentrations of WLR were determined and summarized in Table 1. Slight changes in the IC_50_s in A549 cells were observed among groups, but the changes of the IC_50_s were not dose-dependent, and therefore, they seem to be experimental deviations.

**Table 1:** IC_50_s of oxaliplatin in HCT116 and A549 cancer cells in the absence and presence of WLR treatment

Treatment	IC_50_s (μg/mL)	
	HCT116	A549
L-OHP	1.05 ± 0.07	6.94 ± 0.78
L-OHP + Vehicle	0.97 ± 0.01	5.83 ± 0.02
L-OHP + WLR (10 μg/mL)	0.98 ± 0.06	6.76 ± 0.00
L-OHP + WLR (30 μg/mL)	1.00 ± 0.02	7.66 ± 1.25
L-OHP + WLR (100 μg/mL)	0.98 ± 0.02	6.50 ± 0.30

## Discussion

As a member of the platinum-based chemotherapeutic anticancer drug family, oxaliplatin has widely been used as a key drug to treat patients with advanced and metastatic colorectal cancers as well as other cancer types, such as lung, breast, and ovarian cancers [24]. It is one of the key anticancer drugs because it was chosen to be on a complementary list of the 19^th^ WHO model list of essential medicine (April 2015), category anti-neoplastics and immunosuppressives, subcategory cytotoxic and adjuvant medicine, representing essential medicines for priority diseases [25]. However, oxaliplatin is also known to cause severe and disabling sensory peripheral neuropathy which is a mildly disrupting tingling sensation to extremely painful paresthesia [26]. DRG neurons are a primary target of oxaliplatin-mediated peripheral neurotoxicity because the administered oxaliplatin accumulates and exerts neurotoxicity in the DRG where sensory neurons are located [20]. Acute neuropathy that appears soon after drug administration includes acral paresthesia and dysesthesia which is especially enhanced by exposure to cold. However, chronic neuropathy that appears after repeated administration of the drug leads to dysfunction of sensory and motor neurons [27]. The incidence and severity of chronic neuropathy induced by oxaliplatin in cancer patients can be predicted by those of acute neurological symptoms by oxaliplatin [28]. Oxaliplatin is metabolized to oxalate and platinum metabolite, dichloro (1,2-diaminocyclohexane) platinum [29]. It was known that chronic neuropathy involves morphological damages of the nerve caused by the platinum metabolite of oxaliplatin; however, acute neuropathy caused by oxalate is due to alterations in diverse signaling channels including voltage-gated Ca^2+^, K^+^, and Nav1.9 sodium channels, transient receptor potential-melastatin 8, and -ankyrin 1 channels [13, 27]. According to the severity of the chronic neuropathy the chemotherapeutic dosing paradigm should be modified or delayed, or sometimes, the use of oxaliplatin should be discontinued. Therefore, sustained and chronic neuropathic pain by oxaliplatin is a dose-limiting adverse side effect and may greatly reduce the chemotherapy efficacy as well as quality of life of cancer patients. Unfortunately, however, efficient standard interventions against CIPN including OXPN are not yet clinically available.

In the present study, we demonstrated for the first time that the water extract of *Lithospermi* radix, which has been used as a traditional medicine to treat diverse diseases including acute hepatitis, cancer, and wound healing [30], can reduce the neurotoxicity of oxaliplatin in an NGF-PC12 in vitro model, and it can attenuate the oxaliplatin-induced nociceptive hypersensitivity in an animal model. Our study revealed that twice administration of oxaliplatin at a dose 5 mg/kg (total 10 mg/kg) in C57BL/6 mice that mimics the chronic OXIPN in patients induced enhanced sensitivity to nociceptive mechanical stimuli, and oral administration of WLR can relieve the oxaliplatin-induced mechanical hypersensitivity. We also showed that such mechanical hypersensitivity induced by oxaliplatin is accompanied by activation of both microglia and astrocytes in the dorsal horn of the mouse spinal cords. Astrocytes are related with regulation of CNS inflammation by exerting its diverse cytokines and perivascular microglia supplied from vessels are increased during CNS inflammation and after peripheral nerve damage [31]. Involvement of the activation of spinal glial cells during the development of OXIPN has been previously reported with contradictory results [23, 24, 32–34]. For example, Yoon [32], Robinson [33], and Zheng [34] reported that only spinal astrocytes but not microglias were activated by oxaliplatin treatment. However, Di Cesare Mannelli [23] and Ahn [24] reported that the activation of both spinal microglias and astrocytes was observed following oxaliplatin treatment which is consistent with our results. These contradictory results may be due to the fact that they used different methods to make the oxaliplatin-induced neuropathic animal models, such as the dosing paradigm administered, time of analysis, and choice of experimental animals.

In addition to glial cell activation in the central nerve system, we also observed a significant loss of IENFs in the skin of oxaliplatin-treated C57BL/6 mice. However, the loss of IENF in the skin of foot pad by oxaliplatin was significantly recovered by oral administration of WLR. An IENF is a bare nerve ending comprised of unmyelinated axons from small-diameter sensory neurons [35], and the loss of IENFs in the hands and feet is known to be a consistent pathological finding in patients receiving chemotherapy and experiencing numbness and loss of vibratory senses. Furthermore, loss of IENFs can also be observed in experimental animals by administration of neuropathy-inducing anti-tumor agents including oxaliplatin [14, 36–38]. Xiao et al. reported results similar to ours in a rat OXIPN model showing that repeated administration of low doses of oxaliplatin in rats mimics the clinical development of chronic OXIPN in cancer patients including the induced hypersensitivities in mechanical/cold but not heat stimuli as well as loss of IENFs in the hind paw skin. They also reported the occurrence of atypical swollen and vacuolated mitochondria in peripheral nerve axons by electron microscopy analysis and slowing down of sensory neurons by a nerve conduction study [13]. However, it has not been clearly determined whether the loss of IENFs in the skin is the cause or effect of the changes in neuronal sensitivity following chemotherapy treatment [36].

The precise molecular mechanisms underlying the development of OXIPN remains unclear and should be fully elucidated to prevent or treat established OXIPN. Some research groups suggested that the development of OXIPN involves the activation of the immune system and subsequent induction of inflammation in the satellite cells of the DRG [39] or in the dorsal horn of the spinal cord [36]. Bioenergetic deficits by reactive nitrogen species-mediated inhibition of mitochondrial respiratory complex I and II in peripheral nerve sensory axons [40] and mitochondrial dysfunction by enhanced production of ROS [36] were also suggested as putative mechanisms of OXIPN. Remodeling of ion channel expression in nociceptors such as TREK1 [41], up-regulation of N-methyl-D-aspartate (NMDA) receptor containing subtype 2B (NR2B) and its downstream target, neuronal nitric oxide synthase (nNOS) [27], and enhanced phosphorylation of calcium/calmodulin-dependent protein kinases II [42] were also reported be related with development of OXIPN. It is unambiguous that the development of efficient preventives or therapeutics targeting OXIPN is largely dependent on the understandings of the mechanisms of disease development.

In the present study, we showed that WLR can suppress the spinal activation of microglias and astrocytes and attenuate the mechanical hypersensitivity following oxaliplatin treatment. This finding suggests that WLR may exert an anti-inflammatory activity in neuronal immune cells to attenuate OXIPN. Administration of agents that can ameliorate the neurotoxicity of chemotherapeutics may allow for a stronger chemotherapy regimen like dose escalation. Unfortunately, however, conventional symptomatic management of neuropathic pain such as nortriptyline, amitriptyline, gabapentin, and lamotrigine failed to improve neuropathic patients in placebo-controlled, double-blinded clinical trials [16]. Ca/Mg infusion and venlafaxine, a serotonin-norepinephrine reuptake inhibitor, are known to be effective in preventing OXIPN; however, they are not routinely used because of concerns related with decreased chemotherapy efficacies [43]. Based on the mechanisms underlying the development of OXIPN, several preclinical studies reported some promising agents that exhibited neuroprotection against OXIPN. They include mitoprotective agents like acetyl-L-carnitine and olesosime [13], peroxynitrite scavenger Mn(III) 5, 10, 15, 20-tetrakis (N-n-hexylpyridinium-2-yl) porphyrin [23], immunomodulatory agents minocycline (microglial inhibitor) and fluorocitrate (astrocyte inhibitor) [14, 23], NMDA receptor antagonists KM-801 and memantine or NR2B selective antagonist Ro25-6981 and ifenprodil [27], non-selective NOS inhibitor L-N^G^-nitroarginine methyl ester or nNOS selective inhibitor 7-nitroindazole [27], and glucagon-like peptide-1 agonist exenatide [42]. Such hopeful preclinical findings should be confirmed by prospective randomized, double-blinded, placebo-controlled clinical trials.

When developing supportive drugs to be co-administered with anticancer agents, the interaction between two drugs should be evaluated because a co-administered supportive drug can affect the antitumor activity of anticancer drugs in patients. In the present study, we demonstrated that co-administered WLR did not affect the antitumor activity profiles of oxaliplatin in both human colorectal (HCT116) and non-small cell lung carcinoma (A549) cells. In addition, WLR was revealed not to exert any cytotoxicity in normal cells. Based on this in vitro toxicity study, we expect that WLR can attenuate OXIPN without affecting the anti-tumor effects of oxaliplatin itself and it could be safe for use in preclinical and clinical studies. However, this herb-drug interaction in terms of the anti-tumor effect of oxaliplatin should be confirmed using a suitable xenograft animal model bearing established human cancer cells or patient-derived tumor tissues because we cannot exclude the possibility that co-administered WLR may affect the pharmacokinetics of oxaliplatin in animals.

## Conclusions

In conclusion, our findings show that the extract of medicinal herb WLR can reduce the neurotoxicity of oxaliplatin in vitro and attenuate oxaliplatin-induced mechanical hypersensitivity in an animal model with simultaneous reduction in spinal activation of microglias and astrocytes without affecting the cytotoxicity of oxaliplatin. However, detailed molecular mechanisms underlying the anti-OXIPN potential of WLR and the identity of responsible active constituent(s) of WLR should be elucidated for the development of novel therapeutics using WLR as a starting material.

## Abbreviations

CIPN: Chemotherapy-induced peripheral neuropathy
CMC: Carboxymethylcellulose
CRC: Colorectal cancer
DRG: Dorsal root ganglion
FBS: Fetal bovine serum
GFAP: Glial fibrillary acidic protein
HFFn: Human neonatal foreskin fibroblast
IBA1: Ionized calcium binding adaptor molecule 1
IC_50_: Half maximal inhibitory concentration
IENF: Intraepidermal nerve fibers
NGF: Nerve growth factor
NMDA: N-methyl-D-aspartate
nNOS: Neuronal nitric oxide synthase
NOS: Nitric oxide synthase
NR2B: N-methyl-D-aspartate (NMDA) receptor subtype 2B
OXIPN: Oxaliplatin-induced peripheral neuropathy
PGP9.5: Protein gene product 9.5
ROS: Reactive oxygen species
TNF-α: Tumor necrosis factor-alpha
WLR: Aqueous extract of *Lithospermi* radix
